# Neurofunctional correlates of emotional dysregulation in adolescent Crohn’s disease: a resting-state fMRI preliminary investigation

**DOI:** 10.3389/fnins.2025.1622708

**Published:** 2025-09-02

**Authors:** Ao Liu, Mengting Huang, Xiaoling Deng, Shuo Huang, Keshu Xu

**Affiliations:** ^1^Division of Gastroenterology, Union Hospital, Tongji Medical College, Huazhong University of Science and Technology, Wuhan, China; ^2^Department of Radiology, Union Hospital, Tongji Medical College, Huazhong University of Science and Technology, Wuhan, China; ^3^Hubei Provincial Clinical Research Center for Precision Radiology & Interventional Medicine, Wuhan, China

**Keywords:** adolescent, Crohn’s disease, amplitude of low frequency fluctuations, resting-state functional magnetic resonance imaging, functional connectivity

## Abstract

**Background:**

This study aimed to characterize the relationship between abnormal intrinsic brain function with emotional symptoms in adolescent Crohn’s disease (CD) patients through resting-state functional magnetic resonance imaging (rs-fMRI).

**Methods:**

Forty adolescent CD patient and 53 healthy controls (HCs) were recruited and completed standardized assessments including the Inflammatory Bowel Disease Questionnaire (IBDQ), Symptom Checklist-90 (SCL-90), Social Support Rating Scale (SSRS), and rs-fMRI scans. Compared the intrinsic brain function between groups using amplitude of low-frequency fluctuations (ALFF) and fractional ALFF (fALFF). Subsequent correlation analyses examined relationships between neuroimaging findings clinical indicators, and psychometric measures.

**Results:**

Adolescent CD patients demonstrated significantly lower IBDQ and SSRS scores but higher SCL-90 scores. ALFF abnormalities localized to left superior/inferior temporal gyri, and left precuneus gyrus, while fALFF alterations involved left calcarine fissure and surrounding cortex, Vermis, and left superior frontal gyrus medial. Notably, functional connectivity (FC) of these regions were also changed. Critically, seed-based FC analysis revealed enhanced coupling between left precuneus and both the left superior temporal gyrus and left inferior temporal gyrus and between left medial superior frontal gyrus, left calcarine cortex, and cerebellar vermis, suggesting disease-specific hyperconnectivity in sensory-cognitive networks. Meanwhile, the ALFF in left inferior temporal gyrus was negatively correlated with obsessive compulsive (*r* = −0.348, *p* = 0.028), depression (*r* = −0.344, *p* = 0.003), and anxiety (*r* = −0.388, *p* = 0.013), but positively associated with serum albumin (*r* = 0.338, *p* = 0.033). The fALFF in vermis showed positive association with interpersonal sensitivity (*r* = 0.316, *p* = 0.047), depression (*r* = 0.336, *p* = 0.020), paranoid (*r* = 0.314, *p* = 0.049), psychoticism (*r* = 0.359, *p* = 0.023) in adolescent CD patients.

**Conclusion:**

These findings provide new insights into the neurobiological basis of emotional symptoms in adolescent CD patients, highlighting altered activity in temporal, frontal, and cerebellar regions.

## 1 Introduction

Crohn’s disease (CD), a chronic transmural inflammatory condition of the gastrointestinal tract, frequently manifests during adolescence with distinctive complications with rising global incidence (Vernier-Massouille et al., 2008; Rosen et al., 2015; Piovani et al., 2020; Ng et al., 2017). Adolescents CD patients face unique challenges, including growth retardation, severe disease phenotypes, and psychosocial burdens such as academic disruptions and social isolation (Engel et al., 2021; Turner et al., 2021). Critically, these stressors contribute to a high prevalence of emotional dysregulation, with 5 to 33% of pediatric CD patients developing comorbid mental health’s—a rate significantly exceeding the general population (Stapersma et al., 2018). Population study indicated strong associations between early-onset CD and depression, anxiety, suicidal behaviors, and neurodevelopmental conditions (Butwicka et al., 2019; Mikocka-Walus et al., 2016), while disease-specific factors, such as fatigue (Bevers et al., 2023) and symptom stigma, further impair quality of life (Cooney et al., 2024; Ross et al., 2011). Despite evidence linking CD pathophysiology to gut-brain axis dysregulation, the neurofunctional mechanisms underlying emotional comorbidities in adolescents remain uncharacterized.

Resting-state functional magnetic resonance imaging (rs-fMRI), as a reliable and non-invasive method for assessing brain functions, has become one of the commonly used neuroimaging techniques in brain science research in recent years (Yabluchanskiy et al., 2021). Amplitude of low-frequency fluctuation (ALFF) is an rs-fMRI index used to detect the regional strength of spontaneous fluctuations in Blood Oxygen Level Dependent signals, which can precisely locate spontaneous neural activities in specific brain regions and physiological states (Zang et al., 2007). Changes in ALFF have been reported in CD patients (Huang et al., 2024; Li et al., 2021; Bao et al., 2018). Fractional ALFF (fALFF), derived from ALFF, measures the proportion of low-frequency oscillations within a specific frequency range (typically 0.01–0.08 Hz), thereby representing spontaneous neuronal activity during rest. To a certain extent, it avoids the drawbacks of ALFF, such as being easily affected by cerebrospinal fluid and physiological noise in the ventricle. It can effectively inhibit the non-specific signal components in the resting state fMRI and significantly improve the sensitivity and specificity of local spontaneous brain activity detection (Zou et al., 2008).

Functional connectivity (FC) characterizes the temporal synchronization of spontaneous fluctuations between distinct brain regions, providing insights into the functional integrity of neural networks (Fox and Raichle, 2007). The combined application of these metrics enables comprehensive evaluation of both regional intrinsic activity and disease-associated network alterations. These approaches have been widely employed in functional gastrointestinal disorders and neurological conditions, demonstrating significant potential in resting-state brain function research (Thomann et al., 2025; Langhammer et al., 2025). Consequently, they may prove instrumental in elucidating resting-state neural signatures in adolescents with CD and advancing our understanding of the pathophysiological mechanisms underlying pediatric CD. However, these study cohorts primarily comprised adult CD populations, with observed inconsistencies predominantly confined to adult patients. Specifically, Bao et al. (2018) reported hippocampal hyperactivity in adults, whereas Li et al. (2021) documented hypoactivity in a separate adult cohort. Similarly, Mallio et al. (2020) identified cerebellar connectivity alterations in adults, contrasting with Huang et al.’s (Huang et al., 2024) null findings in another adult sample. These contradictory findings underscore the necessity for further investigation into neural patterns in adolescent CD.

While rs-fMRI has been widely used to investigate neuropsychiatric conditions, no studies to date have systematically examined functional brain alterations in adolescents with CD. Based on emerging evidence of brain-gut interactions in inflammatory bowel disease, we hypothesize that adolescent CD patients exhibit distinct patterns of altered ALFF, fALFF and FC in key brain regions involved in emotion regulation and social cognition. We further hypothesize that these neurofunctional changes correlate significantly with both validated measures of emotional dysregulation and clinically relevant disease characteristics. The primary objective of this study is to examine neural correlates of emotional symptoms changes in adolescent CD patients and explore potential associations between aberrant spontaneous brain activity and emotional symptoms. Additionally, we employed regions exhibiting altered activity in adolescent CD as seed points to map brain functional connectivity patterns using both ALFF and fALFF analyses. This will aid in early intervention and personalized care, and offer a new perspective for guiding the diagnosis, treatment, and prognostic evaluation of the condition.

## 2 Materials and methods

### 2.1 Participants

All adolescent CD patients were consecutively recruited from Department of Gastroenterology, Union Hospital, Tongji Medical College, Huazhong University of Science and Technology between December 2019 to October 2023. Additionally, healthy controls (HCs) were recruited via advertisement. The inclusion criteria of patients were as follows: (1) confirmed diagnosis of CD by a gastroenterologist according to the European Crohn’s and Colitis Organization (Maaser et al., 2019) and the European Society for Pediatric Gastroenterology, Hepatology and Nutrition Diagnostic Criteria (Birimberg-Schwartz et al., 2017); (2) age under 20 years old; (3) right-handed; (4) native language is Chinese; (5) capable of communication and understanding of the survey questionnaires. HCs were recruited according to the following criteria: (1) age- and gender-matched to patients; (2) absence of diagnosed gastrointestinal disorders; and (3) fulfillment of criteria 3–5 identical to those applied to patients.

**Figure 1:**
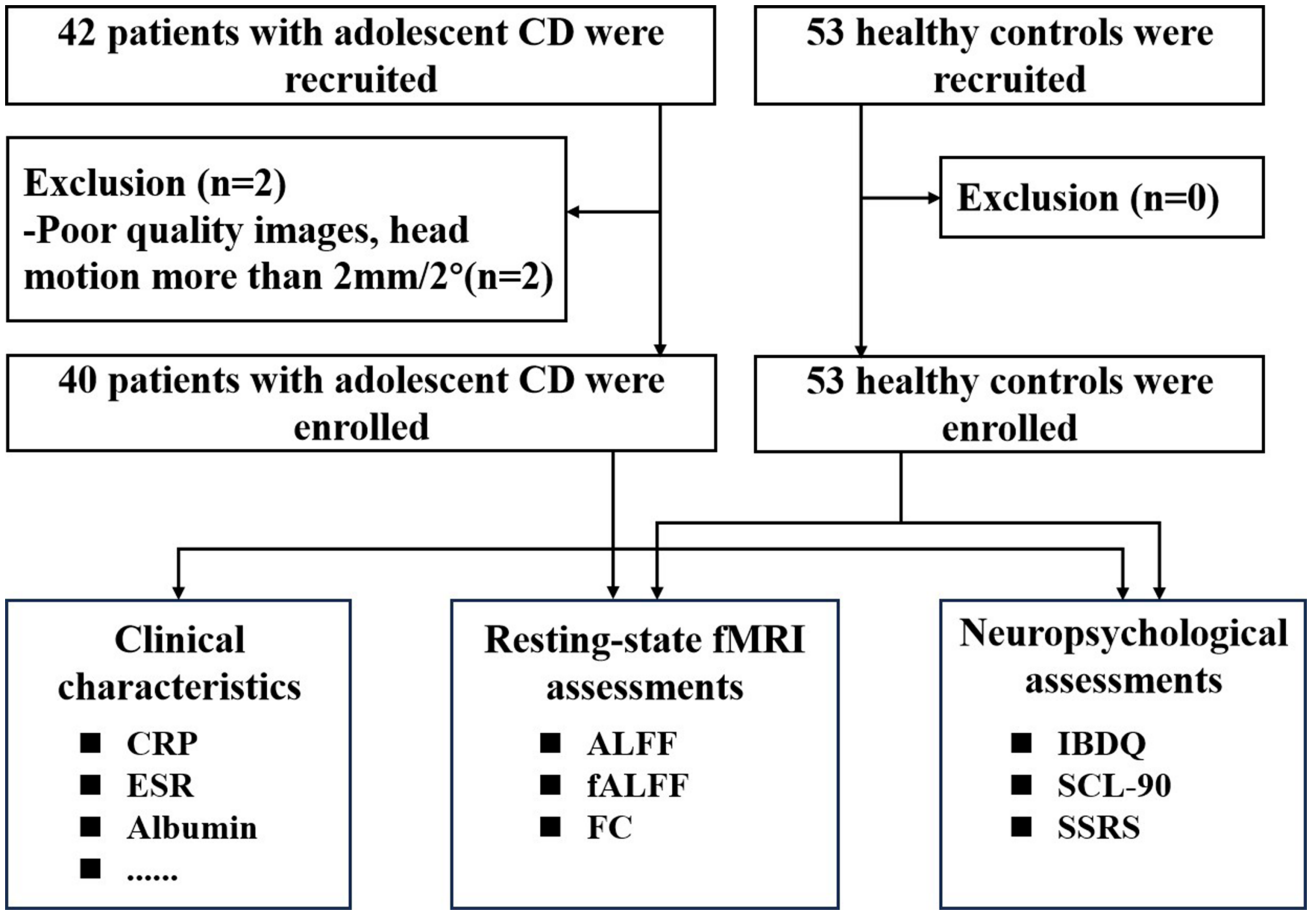
Flow diagram of participant enrollment and assessment. CD, Crohn’s disease, fMRI, functional magnetic resonance imaging; CRP, C-reactive protein; ESR, Erythrocyte sedimentation rate; ALFF, amplitude of low-frequency fluctuation; fALFF, Fractional ALFF; FC,functional connectivity; IBDQ, Inflammatory Bowel Disease Questionnaire; SSRS, Social Support Rating Scale; SCL-90, Symptom Checklist-90.

The exclusion criteria of patients and HCs were as follows: (1) having undergone abdominal surgery related to CD; (2) contraindications for MRI examination; (3) use of psychotropic drugs within the past 3 months; (4) Poor image quality. All patients underwent psychological evaluations and blood sampling to measure inflammatory biomarkers. Following diagnosis and evaluation by gastroenterologists, all participants received monoclonal antibody or anti-inflammatory medication treatment in accordance with good clinical practice. The flow diagram of the enrolled patients with CD and HCs is shown in Figure 1.

This study was approved by the institutional ethics committee of Union Hospital of Tongji Medical College of Huazhong University of Science and Technology (0940–01). All participants were signed informed consent forms.

### 2.2 Clinical and neuropsychological assessments

We collected the clinical and neuropsychological features of a clinical database and neuropsychological questionnaire. The demographic and clinical information includes gender, age, education, site of disease onset, disease duration, serum markers. The Chinese version of the Inflammatory Bowel Disease Questionnaire (IBDQ), the Symptom Checklist-90 (SCL-90) and Social Support Rating Scale (SSRS) were used to assess subjects’ quality of life, anxiety, depression, and other psychological statuses. IBDQ is a validated, disease-specific instrument designed to assess health-related quality of life (HRQoL) in patients with IBD (van den Brink et al., 2018). The IBDQ comprises 32 items across four domains: intestinal symptoms (10 items), systemic symptoms (5 items), social function (5 items), and emotional function (7 items). The SCL-90 and SSRS have been validated in adolescent populations, including those with chronic illnesses (Arnardóttir et al., 2025; Cai et al., 2025; Shi et al., 2023; Benoni et al., 2025; Chu et al., 2025; Riva et al., 2024), as well as in adult patients with CD (Huang et al., 2023). The SCL-90 has shown high reliability in assessing emotional distress in adolescents with chronic conditions (Riva et al., 2024; Zhang et al., 2024), while the SSRS has been used extensively in Chinese adolescent cohorts, demonstrating robust construct validity (Li et al., 2018). Thus, the IBDQ was employed to evaluate HRQoL across all study participants. Concurrently, the SCL-90 was utilized to assess emotional symptoms from multiple dimensions, while the SSRS was administered to measure social support levels among patients. Specific questionnaire contents are provided in Supplementary material 1.

### 2.3 Image acquisition and preprocessing

In this study, a 3.0 T MRI magnetic resonance scanner (SIEMENS SKYRA) was used to acquire functional and anatomical images using a 32-channel head coil. The parameters for the functional imaging were as follows: 240 time points; time of repetition (TR) = 2,000 ms, echo time (TE) = 30 ms, slice thickness = 2.4 mm (no gaps); slices = 60; flip angle (FA) = 90°; field of view (FOV) = 230 × 230 mm; voxel size = 2.4 × 2.4 × 2.4 mm; bandwidth = 1796 Hz/Px, and the total scan time was 8′13″. The parameters for the anatomical imaging were as follows: magnetization prepared rapid gradient echo (MP2RAGE) sequence, TR = 5,000 ms; TE = 2.98 ms, slice thickness = 1.0 mm (no gaps); slices = 176; voxel size = 1 × 1 × 1 mm; FOV = 256 × 240 mm; FA was 9°; bandwidth was 240 Hz/Px; and the total scan time was 8′22″. Before the scan, participants were instructed not to take caffeine containing substances. During the scan, they were asked to lie in a supine position, keep their eyes closed but remain awake. Earplugs and sponge pads were used to reduce scanning noise to protect the participants’ hearing and prevent head movement.

### 2.4 Data preprocessing

The rs-fMRI data were preprocessed using MATLAB R2021a and the DPABI (V7.0) software (Yan et al., 2016). The preprocessing steps were as follows: (1) discarding the first 10 time points, and the data from the remaining 230 time points were retained; (2) slice timing, slice number = 60, slice order = [2:2:60, 1:2:59], reference slice = 60; (3) head motion correction, with over 2.0 mm translation or over 2.0° rotation in any direction were excluded; (4) spatial normalization by the Diffeomorphic Anatomical Registration Through Exponentiated Lie algebra (DARTEL) toolbox (Ashburner, 2007); (5) smooth, with Gaussian kernel of 6 mm full width at half-maximum; and (6) removal of linear trends.

Throughout processing, we adhered to default parameter settings in accordance with established protocols. To ensure data integrity, we implemented a rigorous two-stage quality control protocol. First, all images underwent thorough visual inspection prior to preprocessing to identify and address potential artifacts. Subsequently, post-segmentation statistical quality control was performed to evaluate overall image quality.

### 2.5 ALFF, fALFF calculation and functional connectivity analysis

For ALFF calculation, time series were transformed to the frequency domain via Fast Fourier Transform. Then, the square root of the power spectrum was computed and averaged across the conventional 0.01 to 0.08 Hz band to minimize contamination from high-frequency physiological noise and low-frequency scanner drift. ALFF maps were then converted to z-scores using Fisher’s r-to-z transformation for subsequent group-level analysis.

The fALFF was defined as the ratio of ALFF in the 0.01–0.08 Hz band to the full frequency range (0 to 0.25 Hz). And the fALFF maps underwent identical global mean normalization as ALFF. The fALFF maps were then converted to z-scores using Fisher’s r-to-z transformation for subsequent group-level analysis.

For FC calculation, based on ALFF and fALFF findings, the altered brain regions (voxel size more than 30) between adolescents CD with HCs groups, were chosen as seeds. Spherical regions of interest with 6-mm radius were centered on peak MNI coordinates. Pearson correlation coefficients were computed between each seed region’s mean time course and all other brain voxels. Correlation coefficients underwent Fisher’s z-transformation to improve normality for statistical testing.

### 2.6 Statistical analysis

The differences in demographic and neuropsychological data between the two groups were compared using the two-sample t-test and chi-square test in GraphPad Prism 8 software. A *p*-value less than 0.05 was considered statistically significant. The ALFF, fALFF and FC between the two groups were compared using the two-sample t-test in the statistical module of DPABI, with gender, age, mean FD Jenkinson as covariates. The covariate selection strategy was based on methodological and neurobiological considerations: age and gender were included as standard covariates in neuroimaging studies due to their established effects on resting-state metrics. The head movement parameters (mean FD Jenkinson) was also as one of the covariates for analysis in the subsequent statistics. Then the cluster level correction was performed by Gaussian random field (GRF) correction (voxel *p* < 0.001, cluster *p* < 0.05). The average signal values of brain regions with ALFF and fALFF differences were extracted using DPABI software (Utilities, ROI Signal Extractor). To investigate brain-clinical associations, partial correlation analyses were performed between the neuroimaging markers (ALFF and fALFF) that showed significant group differences and key clinical indicators, as well as neuropsychological data.

## 3 Results

### 3.1 Demographics and clinical characteristics of participants

A total of 40 adolescent CD patients and 53 HCs were included in this study, as shown in Table 1. There were no statistically significant differences between the two groups in terms of demographic characteristics such as age (CD: 18.60 ± 0.98 years, HCs: 18.66 ± 0.71 years, *p* = 0.731), gender (CD: 92.50% male, HCs: 83.02% male, *p* = 0.177), and educational level (CD: 11.95 ± 0.39 years, HCs: 12.00 ± 0.34 years, *p* = 0.511). However, CD patients exhibited significantly lower body mass index (BMI) compared to HCs (CD: 18.99 ± 3.35, HCs: 23.61 ± 1.87, *p* < 0.001). Among CD patients, the mean disease duration was 5.04 ± 8.35 months. Laboratory parameters indicated active inflammation, with elevated erythrocyte sedimentation rate (ESR, 17.20 ± 16.80 mm/h), C-reactive protein (CRP, 16.82 ± 19.49 mg/L), and platelet counts (311.55 ± 131.28 × 10 (Mikocka-Walus et al., 2016)/L). Nutritional parameters showed reduced hemoglobin (149.05 ± 156.90 g/L) and albumin levels (41.22 ± 6.29 g/L). According to the Montreal classification, most patients were diagnosed at age 17 to 20 years (A2: 80.0%), with predominant ileocolonic involvement (L3: 82.5%). Disease behavior was primarily inflammatory (B1: 67.5%), with frequent perianal disease (P: 70.0%).

**Table 1:** Clinical and demographics data of patients with adolescent Crohn’s disease and healthy controls.

Characteristics	CD (*n* = 40)	HCs (*n* = 53)	t/χ^2^	*p* values
Male (n, %)	37 (92.50)	44 (83.02)	1.82	0.177
Age (year)	18.60 ± 0.98	18.66 ± 0.71	0.35	0.731
Body Mass Index	18.99 ± 3.35	23.61 ± 1.87	7.49	<0.001
Education (years)	11.95 ± 0.39	12.00 ± 0.34	0.66	0.511
Duration (month)	5.04 ± 8.35	-	-	-
ESR (mm/h)	17.20 ± 16.80	-	-	-
C-reactive protein (mg/L)	16.82 ± 19.49	-	-	-
Platelet levels (10^9^/L)	311.55 ± 131.28	-	-	-
Hemoglobin (g/L)	149.05 ± 156.90	-	-	-
Albumin (g/L)	41.22 ± 6.29			
Montreal classification	-	-	-	-
A1: A2	8:32			
L1: L2: L3: L4	6:1:33:13	-	-	-
B1: B2: B3: P	27:7:6:28	-	-	-
Current therapy	-	-	-	-
Nutritional therapy (n, %)	4 (10)	-	-	-
5-aminosalicylates (n, %)	7 (17.5)	-	-	-
Biological therapy (n, %)	23 (57.5)	-	-	-
Combined therapy (n, %)	7 (10)	-	-	-

CD, Crohn’s disease; HCs, Healthy controls; ESR, Erythrocyte sedimentation rate; A1, age at diagnosis <16 years; A2, age at diagnosis 17–40 years; L1, ileum; L2, colonic; L3, ileocolonic; L4, upper gastrointestinal tract disease; B1, on inflammatory and no penetrating; B2, inflammatory; B3, penetrating; P, perianal disease.

### 3.2 Neuropsychological characteristics of participants

Comprehensive psychological assessments revealed significant differences between adolescent CD patients and HCs across multiple domains (Table 2). Quality of life measures demonstrated that CD patients had significantly worse bowel symptoms (*p* < 0.001), emotional function (*p* = 0.006), and social function (*p* < 0.001), resulting in a lower total IBDQ score (*p* < 0.001). Social support evaluation showed CD patients had significantly reduced objective support (*p* = 0.008), subjective support (*p* < 0.001), and support availability (*p* = 0.031), with a consequently lower total SSRS score (*p* < 0.001).

**Table 2:** Psychological characteristics of patients with adolescent Crohn’s disease.

Scale	Characteristic	Total (*n* = 93)	CD (*n* = 40)	HCs (*n* = 53)	*p* values
IBDQ	Bowel symptoms	61.23 ± 7.87	57.52 ± 9.17	64.02 ± 5.29	<0.001^*^
	Systemic symptoms	27.02 ± 4.45	26.57 ± 5.35	27.36 ± 3.65	0.429
	Emotional function	68.23 ± 8.72	65.22 ± 10.00	70.49 ± 6.88	0.006^*^
	Social function	30.46 ± 5.52	26.60 ± 5.99	33.38 ± 2.60	<0.001^*^
	Total IBDQ	186.94 ± 23.64	175.93 ± 27.77	195.25 ± 15.71	<0.001^*^
SSRS	Objective support	8.37 ± 2.27	7.65 ± 1.79	8.91 ± 2.46	0.008^*^
	Subjective support	18.90 ± 4.68	16.48 ± 4.91	20.74 ± 3.56	<0.001^*^
	Availability	7.75 ± 1.67	7.33 ± 1.85	8.08 ± 1.45	0.031^*^
	Total SSRS	35.02 ± 6.43	31.45 ± 6.68	37.72 ± 4.74	<0.001^*^
SCL-90	Somatization	14.94 ± 4.66	16.65 ± 5.71	13.64 ± 3.14	0.004^*^
	Obsessive compulsive	15.08 ± 5.10	16.43 ± 5.37	14.06 ± 4.68	0.026^*^
	Interpersonal sensitivity	12.37 ± 4.42	14.43 ± 5.30	10.81 ± 2.77	<0.001^*^
	Depression	17.23 ± 5.96	19.45 ± 7.13	15.55 ± 4.25	0.003^*^
	Anxiety	12.70 ± 4.12	13.95 ± 5.00	11.75 ± 3.03	0.017^*^
	Hostility	7.91 ± 3.16	9.25 ± 3.83	6.91 ± 2.08	<0.001^*^
	Phobic anxiety	8.16 ± 2.37	8.88 ± 2.91	7.62 ± 1.70	0.018^*^
	Paranoid	7.33 ± 2.54	8.20 ± 3.19	6.68 ± 1.66	0.004^*^
	Psychoticism	12.04 ± 3.66	13.43 ± 4.44	11.00 ± 2.50	0.003^*^
	Other	9.14 ± 2.67	10.35 ± 2.90	8.23 ± 2.07	<0.001^*^
	Total SCL90	116.89 ± 34.51	131.00 ± 40.62	106.25 ± 24.51	0.001^*^

CD, Crohn’s disease; HCs, healthy controls; IBDQ, Inflammatory Bowel Disease Questionnaire; SSRS, Social Support Rating Scale; SCL-90, Symptom Checklist-90. ^*^Indicates a significant difference between CD group and HCs group (*p* < 0.05); for each factor, we calculated the mean score (sum of item scores divided by the number of items per factor).

Psychological symptom profiles using the SCL-90 revealed CD patients scored significantly higher across all subscales: somatization (*p* = 0.004), obsessive-compulsive symptoms (*p* = 0.026), interpersonal sensitivity (*p* < 0.001), depression (*p* = 0.003), anxiety (*p* = 0.017), hostility (*p* < 0.001), phobic anxiety (*p* = 0.018), paranoid ideation (*p* = 0.004), psychoticism (*p* = 0.003), and other symptoms (*p* < 0.001). These differences culminated in a significantly higher total SCL-90 score for CD patients (*p* = 0.001). These findings demonstrate substantial psychological burden in adolescent CD patients, characterized by impaired quality of life, reduced social support, and elevated psychological symptoms scores across multiple symptom domains. Neuropsychological assessments showed that there were statistically significant differences in multiple indicators between the two groups (*p* < 0.05), as shown in Table 2. Overall, compared with the HCs, adolescent CD patients had lower scores on the IBDQ and SSRS, and higher scores on the SCL-90, indicating that adolescent CD patients had emotional symptoms of varying degrees.

### 3.3 ALFF and fALFF results

**Figure 2:**
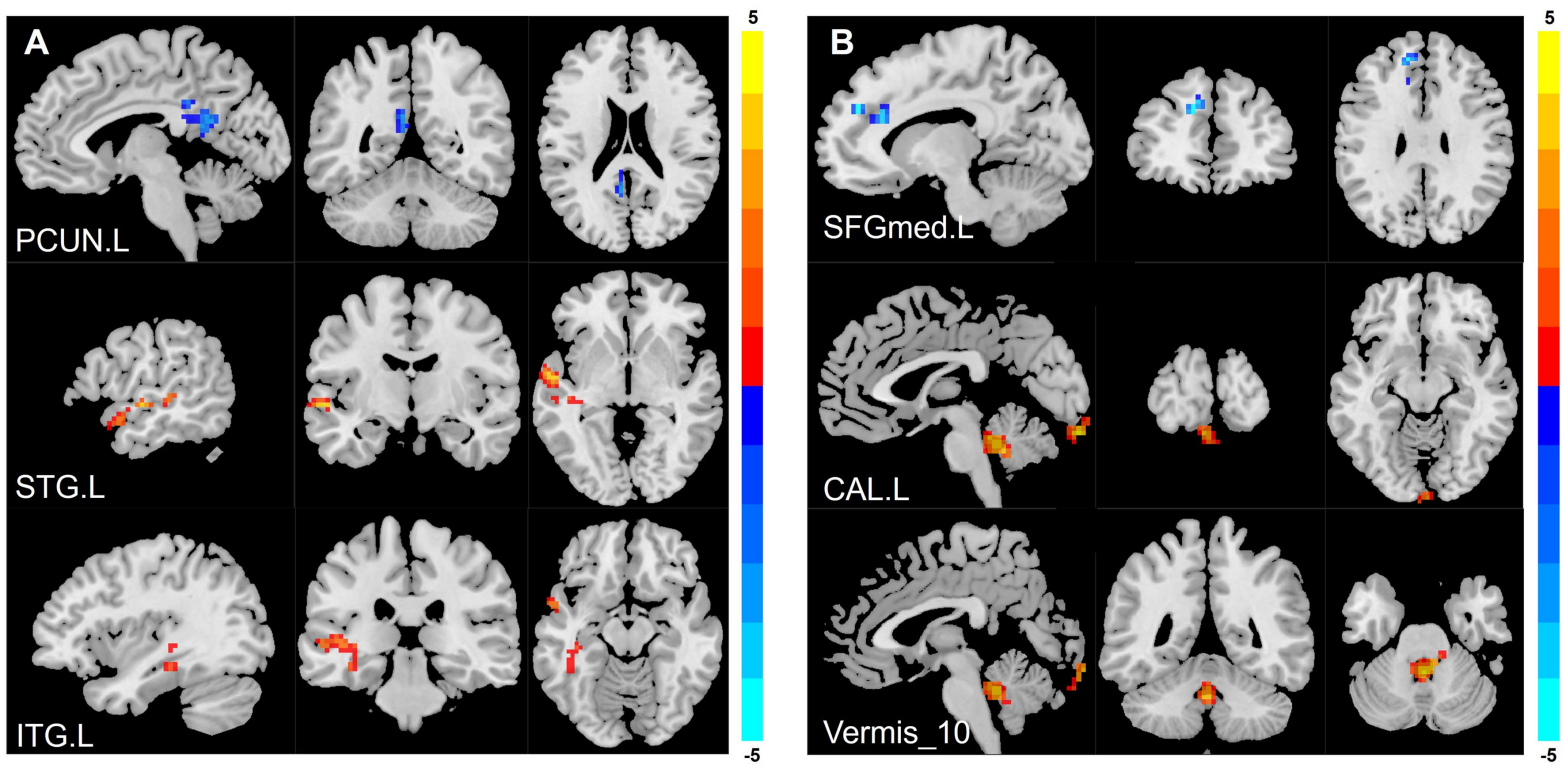
Whole-brain maps of ALFF and fALFF differences between adolescent CD patients and healthy controls. **(A)** Amplitude of low-frequency fluctuation (ALFF) differences: Increased activity (red) in left superior/inferior temporal gyri (STG. L/ITG. L); decreased activity (blue) in left precuneus (PCUN. L). **(B)** Fractional ALFF (fALFF) differences: Increased activity (red) in left calcarine cortex (CAL. L) and cerebellar vermis (Vermis_10); decreased activity (blue) in left medial superior frontal gyrus (SFGmed. L). The color bar represents the t-value.

**Figure 3:**
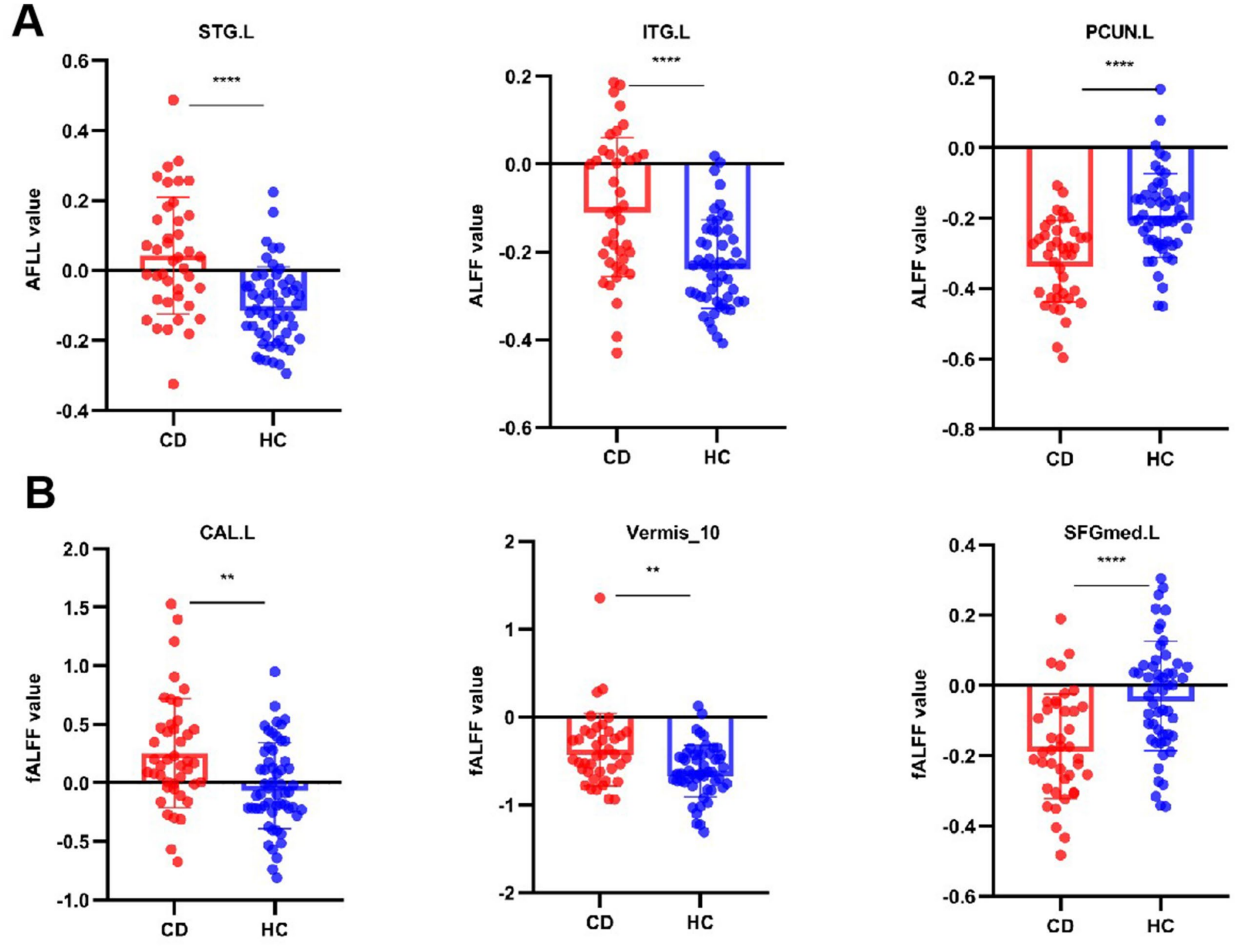
Quantitative comparison of ALFF and fALFF values in regions showing significant group differences. Bar graphs compare adolescents with Crohn’s disease (CD) and healthy controls (HCs) for: **(A)** Amplitude of low-frequency fluctuation (ALFF) in left superior/inferior temporal gyri (STG. L/ITG. L), and left precuneus (PCUN. L); **(B)** Fractional ALFF (fALFF) in left calcarine cortex (CAL. L), cerebellar vermis (Vermis_10), and left medial superior frontal gyrus (SFGmed. L). **p < 0.01, ***p < 0.001 (two-sample *t*-test).

Compared to the HCs, adolescent CD group showed higher ALFF values in left superior temporal gyrus (STG. L) and left inferior temporal gyrus (ITG. L), and showed lower ALFF values in left precuneus gyrus (PCUN. L). Compared to the HCs, adolescent CD group showed higher fALFF values in left calcarine cortex (CAL. L) and cerebellar vermis (Vermis 10), and showed lower fALFF values in left superior frontal gyrus, medial (SFGmed. L). The detailed descriptions of brain regions were shown in Table 3 and Figure 2. We used the Harvard-Oxford cortical and subcortical structural atlases and the AAL3 template to identify regions. The mean values extracted from the brain regions with differences in ALFF and fALFF are presented in Figure 3.

**Table 3:** Brain regions with significant differences in amplitude of low frequency fluctuations between patients with adolescent Crohn’s disease and healthy controls.

Regions	X	Y	Z	Voxel size	T value
ALFF: CD > HCs					
STG. L	−57	−9	−3	54	4.682
ITG. L	−38	−28	−13	81	3.707
ALFF: CD < HCs					
PCUN. L	−6	−51	21	55	−4.636
fALFF: CD > HCs					
CAL. L	−2	−93	−11	54	4.712
Vermis_10	2	−47	−29	79	3.701
fALFF: CD < HCs					
SFGmed. L	−12	45	30	36	−4.508

CD, Crohn’s disease; HCs, healthy controls; ALFF, amplitude of low-frequency fluctuation, fALFF, Fractional ALFF; STG. L, left superior temporal gyrus; PCUN. L, left precuneus gyrus; ITG. L, left inferior temporal gyrus; CAL. L, left calcarine cortex; Vermis_10, cerebellar vermis; SFGmed. L, left medial superior frontal gyrus. The statistical threshold was set at voxel *p* < 0.001, cluster *p* < 0.05, and two-tailed (GRF correction). X, Y, and Z coordinates were the primary peak locations in the MNI space.

**Figure 4:**
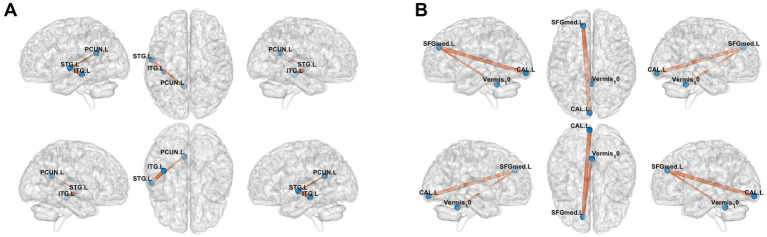
Results of functional connectivity analysis selecting the mask of ALFF and fALFF as seed point. **(A)** Significant brain regions of amplitude of low-frequency fluctuation (ALFF) showing increased functional connectivity of the left superior/inferior temporal gyri (STG. L/ITG. L) and left precuneus (PCUN. L) between adolescent Crohn’s disease and healthy controls. **(B)** Significant brain regions of Fractional ALFF (fALFF) showing increased functional connectivity of the left calcarine cortex (CAL. L), cerebellar vermis (Vermis_10), and left medial superior frontal gyrus (SFGmed. L). Orange regions indicate hyperconnectivity in CD patients compared to HCs.

Seed-based FC analysis revealed distinct patterns of altered neural connectivity in adolescent CD patients compared to HCs. The ALFF-derived connectivity maps (Figure 4A) demonstrated significantly enhanced functional coupling between the PCUN. L and both the STG. L and ITG. L, as evidenced by prominent orange connectivity pathways. Similarly, fALFF-based analysis (Figure 4B) showed increased connectivity involving the SFGmed. L, CAL. L, and Vermis_10, with robust interregional connectivity visualized at coordinates. These findings collectively suggest widespread functional reorganization in CD patients, particularly affecting networks integrating higher-order cognitive (PCUN. L-SFGmed. L), sensory (STG. L-ITG. L), and visual-cerebellar (CAL. L-Vermis 10) processing systems. These findings suggest disease-specific alterations in both cognitive-limbic and visual-cerebellar neural circuits.

### 3.4 Correlation among clinical data, neuropsychological data, and ALFF and fALFF values

**Figure 5:**
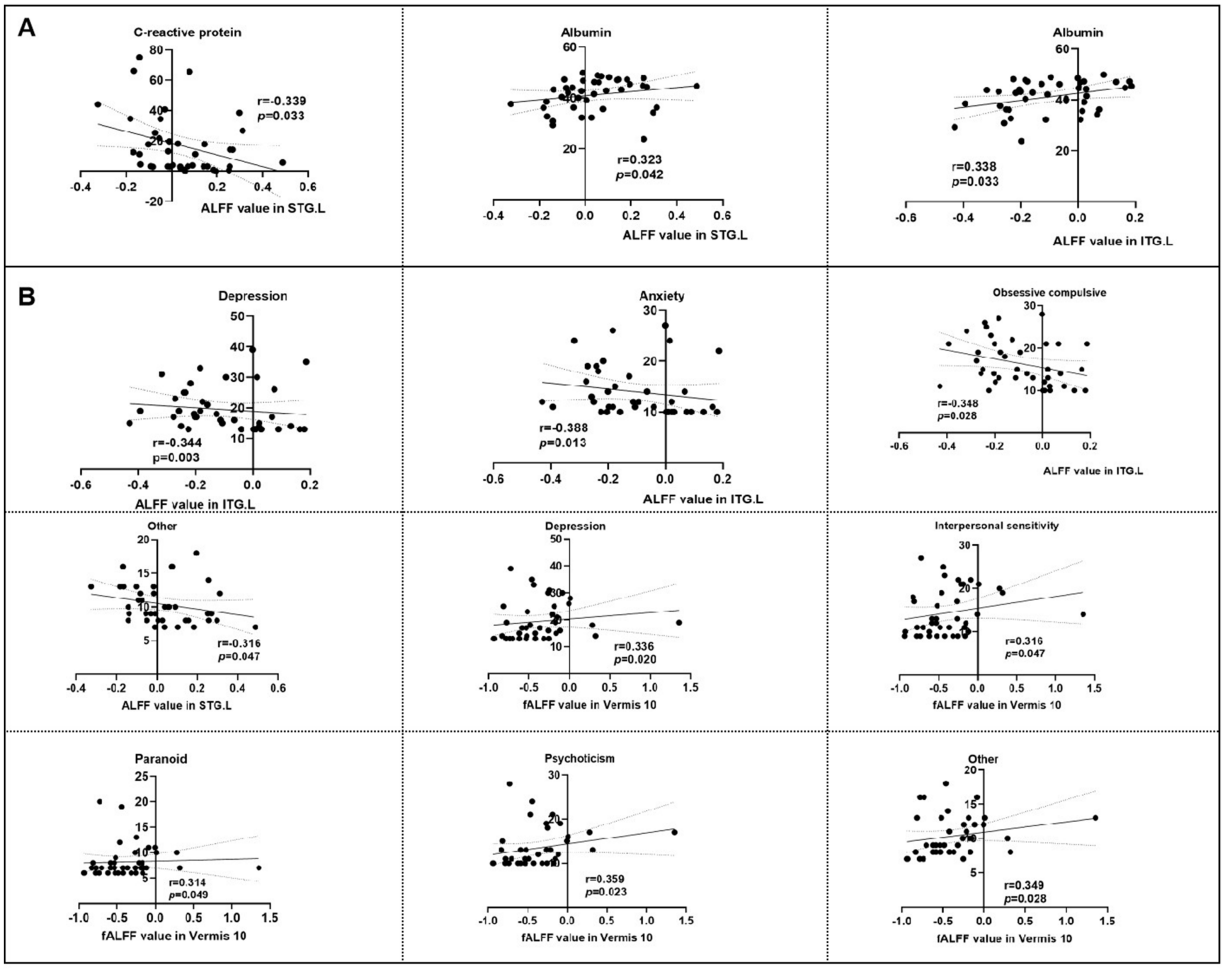
Correlation analysis of intrinsic brain activity with neuropsychological scores and clinical information. ALFF, amplitude of low-frequency fluctuation, fALFF, Fractional ALFF; STG. L, left superior temporal gyrus; PCUN. L, left precuneus gyrus; ITG. L, left inferior temporal gyrus. **(A)** Correlation analysis of intrinsic brain activity with clinical information. **(B)** Correlation analysis of intrinsic brain activity with neuropsychological scores.

Significant correlations emerged between aberrant neural activity and clinical metrics. As revealed in Figure 5A, the ALFF values in the STG. L was negatively correlated with the CRP (*r* = −0.339, *p* = 0.033), but positive correlation with albumin (*r* = 0.323, *p* = 0.042). The ALFF value in ITG. L was positive correlation with albumin (*r* = 0.338, *p* = 0.033). Meanwhile, the psychological symptomatology of SCL-90 score is also significantly correlated with the neural activity. Specifically, the ALFF value in ITG. L was positive negatively with SCL-90 sub-scores for depression scores (*r* = −0.344, *p* = 0.003), anxiety scores (*r* = −0.388, *p* = 0.013), obsessive compulsive scores (*r* = −0.348, *p* = 0.028), and the ALFF value in STG. L was negatively correlated with other scores (*r* = −0.316, *p* = 0.047; Figure 5B). However, the fALFF in Vermis 10 region was positive correlation with depression scores (*r* = 0.336, *p* = 0.020), interpersonal sensitivity scores (*r* = 0.316, *p* = 0.047), paranoid scores (*r* = 0.314, *p* = 0.049), psychoticism scores (*r* = 0.359, *p* = 0.023), and other scores (*r* = 0.349, *p* = 0.028) in SCL90 (Figure 5B).

## 4 Discussion

Our study demonstrates that adolescents with CD exhibit significantly lower IBDQ and SSRS scores compared to HCs, while demonstrating significantly higher SCL-90 scores. Abnormal ALFF values are found in the left superior temporal gyrus, left inferior temporal gyrus, and left precuneus gyrus. Abnormal fALFF values were identified in the left calcarine fissure and surrounding cortex, vermis, and the left superior frontal gyrus, medial of the brain regions in adolescent CD patients. Furthermore, ALFF and fALFF value exhibited significant correlations with intestinal inflammation and nutritional indicators, and emotional symptoms scores in adolescent CD patients.

Our study found that adolescents with CD exhibited significantly impaired quality of life and diminished social support relative to HCs, concurrently presenting with elevated emotional symptom scores. These findings are consistent with previous research on adult CD patients (Huang et al., 2023), emphasizing the multifaceted nature of CD. Previous studies have shown that male and IBD patients aged 12 to 17 are at the highest risk of developing mental health conditions (McCartney et al., 2022), and children IBD patients are associated with higher prevalence of anxiety and depression (Loftus et al., 2011). The decline in quality of life and social support may stem from the unique behavioral issues associated with adolescent CD (Griffiths, 2004), such as perianal disease, abdominal pain, and fatigue (Greenley et al., 2010; Mackner et al., 2013). The embarrassing nature of IBD symptoms (such as frequent bowel movements, diarrhea etc.) can lead to social isolation, strained family relationships (Cushman et al., 2020), and academic difficulties among adolescent patients with CD, all of which contribute to a decrease in overall quality of life. Higher emotional symptoms scores, including symptoms of obsessive compulsive, depression, and anxiety, indicate that adolescent CD often coexists with other mental health conditions. This comorbidity can further complicate the treatment and management of adolescent CD, as addressing one aspect may require a comprehensive consideration of the interplay between behavioral and emotional symptoms.

Most prior studies included adult CD patients in remission or active stages, whereas our study focuses on adolescents, a population with unique neurodevelopmental and psychosocial challenges. In terms of resting-state ALFF and fALFF, we found that the ALFF values in the left precuneus gyrus, left superior temporal gyrus, and left inferior temporal gyrus were abnormally altered in the adolescent CD group. Reduced ALFF in the left precuneus aligns with Li et al. (2021), suggesting a consistent role of the default mode network in CD-related emotional dysregulation. The fALFF values were changed in the left calcarine fissure and surrounding cortex, left superior frontal gyrus, medial, and Vermis brain regions. The temporal gyrus is involved in auditory processing, language comprehension (Gernsbacher and Kaschak, 2003), social cognition (Jou et al., 2010), memory and learning (Jou et al., 2010), all of which are crucial for normal social interaction. Early-onset CD may confer greater neurodevelopmental risk, as inflammation during puberty could disrupt limbic system maturation. The relatively limited sample size in the age less than 16 years subgroup may constrain certain age-stratified analyses. However, we incorporated age as a covariate in all comparative analyses of ALFF and fALFF parameters between CD patients and healthy controls, thereby effectively controlling for potential confounding effects of age-related variation.

Increased ALFF in the left superior and inferior temporal gyri is a novel finding. These regions mediate auditory processing (Sun et al., 2025), language comprehension (Xu et al., 2020), and social cognition (Li et al., 2025), which may explain the social withdrawal and interpersonal sensitivity observed in adolescent CD patients. Abnormal ALFF values in the left superior temporal gyrus and left inferior temporal gyrus may disrupt these functions, thereby leading to social and behavioral deficits in patients with adolescent CD (Gamwell et al., 2018). The precuneus gyrus is associated with self-awareness processing and episodic memory (Cavanna and Trimble, 2006), which may be related to the self-regulation and emotional dysregulation issues commonly seen in adolescent CD. The left calcarine fissure and surrounding cortex are associated with visual processing, while the Vermis of the cerebellum is involved in motor control and cognitive functions (Devita et al., 2025; Chao et al., 2023). Although research on the function of the Vermis is relatively limited at present, as part of the cerebellar vermis, it has become a key node in the integrated network that connects different functions such as sensory, motor, emotional, and cognitive functions (Bostan and Strick, 2018; Stoodley and Tsai, 2021). While not directly mediating gastrointestinal function, dysregulation in visual and cerebellar networks may amplify symptom awareness and compromise emotional-motor adaptation, thereby contributing to the subjective burden of CD. Abnormal fALFF values in these regions may indicate a more widespread disruption of the neural networks that integrate sensory, motor, and cognitive processes, which may underlie the complex behavioral and emotional symptoms of adolescent CD. These findings provide new insights into the neurobiological basis of adolescent CD patients. Prior adult studies have not emphasized temporal lobe alterations, possibly due to age-related neural plasticity differences. The current results highlight age-dependent changes in brain regions critical for emotional regulation (e.g., temporal gyrus) and self-awareness (precuneus), whereas adult studies focus more on sensorimotor and frontal lobe networks. This underscores the need for age-stratified CD neuroimaging research. The discrepancy between adult and adolescent ALFF patterns suggests that CD-related brain alterations may emerge during critical neurodevelopmental stages, influencing long-term emotional comorbidity risk.

In this study, the ALFF-derived connectivity maps demonstrated significantly enhanced functional coupling between the PCUN. L and both STG. L and ITG. L. The PCUN. L is a hub of the default mode network (DMN), governing self-referential processing and episodic memory. Its hyperconnectivity with STG. L and ITG. L—regions critical for social cognition and auditory-emotional integration (Gernsbacher and Kaschak, 2003)—suggests maladaptive coupling between self-focused rumination and socioemotional processing. This may underpin excessive symptom monitoring and interpersonal sensitivity observed in CD adolescents. Meanwhile, the fALFF-derived connectivity maps demonstrated significantly enhanced functional coupling between the SFGmed. L and both CAL. L, and Vermis_10. The SFGmed. L supports executive control and emotion regulation (Devita et al., 2025). Its hyperconnectivity with visual cortex and cerebellar vermis—a region implicated in visceromotor and affective modulation (Stoodley and Tsai, 2021)—indicates dysregulated integration of sensory input with top-down cognitive control, potentially contributing to anxiety and somatic preoccupation. Moreover, FC in these brain regions has also been altered in adolescent CD patients, which is partly consistent with previous studies (Mallio et al., 2020; Thapaliya et al., 2023). This suggests that changes in underlying neural activity (reflected by ALFF and fALFF) may potentially drive the observed FC changes. Alterations in FC across multiple brain regions may manifest as heightened sensitivity to visceral sensory information (Langhammer et al., 2025), such as increased symptom monitoring, heightened vigilance, and anxiety about anticipated abdominal pain, cramps, and diarrhea.

The ALFF and fALFF indices in these brain regions are correlated with emotional symptoms scores, which also illustrate this point to some extent. In adolescent CD patients, the abnormal increase in FC in these regions may indicate compensatory mechanisms for underlying neuropathology. Understanding these relationships between neural activity and connectivity is crucial for deciphering the complex pathophysiology of CD. The hyperconnectivity between DMN nodes and social-cognitive regions may amplify salience attribution to visceral sensations, fostering catastrophic appraisals of symptoms. Concurrently, weakened connectivity between SFGmed. L and cerebellar vermis could impair downregulation of limbic arousal. Our study identifies ALFF values increased in left superior temporal gyrus and inferior temporal gyrus, but decreased in left precuneus gyrus. Reduced ALFF in the left precuneus aligns with Li et al. (2021), while Bao et al. (2018) reported default mode network alterations encompassing this region, suggesting a consistent role of the DMN in CD-related emotional dysregulation.

Furthermore, our correlation analyses revealed clinically meaningful associations between aberrant neural activity and both emotional symptoms and disease biomarkers. Specifically, the ALFF value in the ITG. L demonstrated a negative correlation with obsessive-compulsive symptoms, depression, and anxiety, while exhibiting a positive correlation with serum albumin levels. Similarly, the ALFF in the STG. L was negatively correlated with CRP and positively correlated with albumin. These findings suggest that increased inflammation (reflected by elevated CRP) and malnutrition (indicated by hypoalbuminemia) may contribute to dysregulated neural activity in temporal regions involved in social cognition and auditory processing. Conversely, reduced ALFF in these regions correlates with heightened psychopathological burden, reinforcing the role of the temporal cortex in mediating gut-brain interactions in adolescent CD. Inflammation in the gut may release cytokines and other immune mediators that can cross the blood brain barrier and affect neural function (Agirman et al., 2021). Additionally, medications used in CD can modulate inflammatory markers and brain activity, which were not controlled for in this cross-sectional design. Future longitudinal studies with detailed inflammatory profiling are needed to disentangle the specific contributions of gut-derived inflammation to neural alterations. Collectively, these results position the temporal gyri as critical neural interfaces bridging gastrointestinal pathophysiology and behavioral comorbidities, potentially implicating neuroinflammatory pathways in the gut-brain axis. The positive correlations between fALFF in Vermis and emotional symptoms scores, including interpersonal sensitivity, depression, paranoid, and psychoticism, further emphasize the link between brain function and emotional well-being in CD patients. The cerebellum is increasingly recognized for its role in emotional regulation and cognitive processing (Turner et al., 2007). This relationship also highlights the potential for targeting the cerebellum in the development of novel therapeutic interventions for CD, such as non-invasive brain stimulation techniques.

There are still some limitations of our study. Firstly, the cross-sectional design precludes temporal inferences; longitudinal investigations are required to delineate the evolution of observed changes. Secondly, our cohort exhibited pronounced male predominance, and heterogeneous pharmacotherapy regimens initiated prior to enrollment were present. These pharmacological interventions may confound normative cerebral metabolism through diverse pathways, consequently exerting differential effects on neurofunctional activity. Third, while we selected well-validated measures for adolescent populations, the IBDQ’s original validation in adolescent requires cautious interpretation of quality-of-life metrics in this transitional age group. Our findings reflect subclinical emotional symptoms measured by SCL-90, not diagnosed psychiatric symptoms. Future studies should incorporate structured clinical interviews to assess symptoms comorbidity. Methodologically, seed-based FC analyses identify temporal correlations between neural regions but cannot establish directional influences. Finally, the modest sample size inherently constrains statistical power to detect subtle neurofunctional alterations and precludes robust subgroup analyses (e.g., active vs. quiescent disease, depression comorbidity status) that might elucidate phenotype-specific neural signatures. Future expanded longitudinal cohorts, stratified by sex and medication status, should track neurofunctional trajectories alongside molecular profiling to establish mechanistic links between gut inflammation and neural circuit dysregulation.

## 5 Conclusion

In conclusion, our study found that adolescent CD patients showed altered emotional symptoms, and their intrinsic brain function, which may relate to behavioral issues, was also affected. Future research should focus on exploring the underlying mechanisms, and developing targeted interventions based on these insights.

## Data Availability

The original contributions presented in the study are included in the article/Supplementary material, further inquiries can be directed to the corresponding authors.
