# Supplementary material for: Neurofunctional correlates of emotional dysregulation in adolescent Crohn’s disease: a resting-state fMRI preliminary investigation
Source: Front Neurosci. 2025 Sep 2;19:1622708. doi: 10.3389/fnins.2025.1622708 (PMC12436501; doi:10.3389/fnins.2025.1622708)
Supplement: Supplementary file 1 [file Table_1.DOCX]

Supplementary Material

# Supplementary Material 1

**The Chinese version of the Inflammatory Bowel Disease Questionnaire (IBDQ).**

This questionnaire assesses your symptoms and quality of life over the past 2 weeks. There are 32 questions, each with responses scored from 1 to 7. Please select the option that best reflects your experience.

1. Over the past 2 weeks, how frequent have your bowel movements been?

① More frequent than usual or as severe as ever before; ② Extremely frequent; ③ Very frequent; ④ Moderately increased frequency; ⑤ Slightly increased frequency; ⑥Minimally increased frequency; ⑦ Normal frequency (no increase)

2. Over the past 2 weeks, how much time have you spent feeling fatigued, weak, or exhausted?

① All the time; ② Most of the time; ③ A lot of the time; ④ Some of the time; ⑤ A little of the time; ⑥ Rarely; ⑦ Not at all

3. Over the past 2 weeks, how much time have you spent feeling frustrated, impatient, or irritable?

① All the time; ② Most of the time; ③ A lot of the time; ④ Some of the time; ⑤ A little of the time; ⑥ Rarely; ⑦ Not at all

4. Over the past 2 weeks, how much time have you missed school or work due to bowel problems?

① All the time; ② Most of the time; ③ A lot of the time; ④ Some of the time; ⑤ A little of the time; ⑥ Rarely; ⑦ Not at all

5. Over the past 2 weeks, how much time have you had loose stools?

① All the time; ② Most of the time; ③ A lot of the time; ④ Some of the time; ⑤ A little of the time; ⑥ Rarely; ⑦ Not at all

6. Over the past 2 weeks, how would you rate your energy level?

① No energy at all; ② Very little energy; ③ Slight energy; ④ Some energy; ⑤ Moderate energy; ⑥ A lot of energy; ⑦ Abundant energy

7. Over the past 2 weeks, how much time have you worried about needing surgery for your bowel condition?

① All the time; ② Most of the time; ③ A lot of the time; ④ Some of the time; ⑤ A little of the time; ⑥ Rarely; ⑦ Not at all

8. Over the past 2 weeks, how much time have you delayed or canceled social activities due to bowel problems?

① All the time; ② Most of the time; ③ A lot of the time; ④ Some of the time; ⑤ A little of the time; ⑥ Rarely; ⑦ Not at all

9. Over the past 2 weeks, how much time have you been troubled by abdominal cramps?

① All the time; ② Most of the time; ③ A lot of the time; ④ Some of the time; ⑤ A little of the time; ⑥ Rarely; ⑦ Not at all

10. Over the past 2 weeks, how much time have you felt physically unwell?

① All the time; ② Most of the time; ③ A lot of the time; ④ Some of the time; ⑤ A little of the time; ⑥ Rarely; ⑦ Not at all

11. Over the past 2 weeks, how much time have you been troubled by worrying about finding a toilet?

① All the time; ② Most of the time; ③ A lot of the time; ④ Some of the time; ⑤ A little of the time; ⑥ Rarely; ⑦ Not at all

12. Over the past 2 weeks, how much difficulty did bowel problems cause in your desired leisure or sports activities?

① Severe difficulty (unable to participate); ② Considerable difficulty; ③ Moderate difficulty; ④ Some difficulty; ⑤ Little difficulty; ⑥ Minimal difficulty; ⑦ No difficulty

13. Over the past 2 weeks, how much time have you been troubled by abdominal pain?

① All the time; ② Most of the time; ③ A lot of the time; ④ Some of the time; ⑤ A little of the time; ⑥ Rarely; ⑦ Not at all

14. Over the past 2 weeks, how much time have you been troubled by inability to sleep or waking up at night?

① All the time; ② Most of the time; ③ A lot of the time; ④ Some of the time; ⑤ A little of the time; ⑥ Rarely; ⑦ Not at all

15. Over the past 2 weeks, how much time have you felt depressed or despondent?

① All the time; ② Most of the time; ③ A lot of the time; ④ Some of the time; ⑤ A little of the time; ⑥ Rarely; ⑦ Not at all

16. Over the past 2 weeks, how much time have you been unable to go to places due to the absence of nearby toilets?

① All the time; ② Most of the time; ③ A lot of the time; ④ Some of the time; ⑤ A little of the time; ⑥ Rarely; ⑦ Not at all

17. Overall, how much of a problem was excessive flatulence for you over the past 2 weeks?

① A severe problem; ② A major problem; ③ A noticeable problem; ④ Somewhat bothersome; ⑤ Slightly bothersome; ⑥ Rarely bothersome; ⑦ Not a problem

18. Overall, how much of a problem was maintaining or achieving your desired weight over the past 2 weeks?

① A severe problem; ② A major problem; ③ A noticeable problem; ④ Somewhat bothersome; ⑤ Slightly bothersome; ⑥ Rarely bothersome; ⑦ Not a problem

19. Overall, how much time have you spent worrying about your bowel condition (e.g., cancer risk, disease recurrence) over the past 2 weeks?

① All the time; ② Most of the time; ③ A lot of the time; ④ Some of the time; ⑤ A little of the time; ⑥ Rarely; ⑦ Not at all

20. Over the past 2 weeks, how much time have you been troubled by abdominal bloating?

① All the time; ② Most of the time; ③ A lot of the time; ④ Some of the time; ⑤ A little of the time; ⑥ Rarely; ⑦ Not at all

21. Over the past 2 weeks, how much time have you felt relaxed and stress-free?

① All the time; ② Most of the time; ③ A lot of the time; ④ Some of the time; ⑤ A little of the time; ⑥ Rarely; ⑦ Not at all

22. Over the past 2 weeks, how much time have you had rectal bleeding?

① All the time; ② Most of the time; ③ A lot of the time; ④ Some of the time; ⑤ A little of the time; ⑥ Rarely; ⑦ Not at all

23. Over the past 2 weeks, how much time have you felt embarrassed by your bowel problems?

① All the time; ② Most of the time; ③ A lot of the time; ④ Some of the time; ⑤ A little of the time; ⑥ Rarely; ⑦ Not at all

24. Over the past 2 weeks, how much time have you been troubled by the urge to defecate despite an empty bowel?

① All the time; ② Most of the time; ③ A lot of the time; ④ Some of the time; ⑤ A little of the time; ⑥ Rarely; ⑦ Not at all

25. Over the past 2 weeks, how much time have you felt sad, tearful, or emotionally upset?

① All the time; ② Most of the time; ③ A lot of the time; ④ Some of the time; ⑤ A little of the time; ⑥ Rarely; ⑦ Not at all

26. Over the past 2 weeks, how much time have you been troubled by accidental underwear soiling?

① All the time; ② Most of the time; ③ A lot of the time; ④ Some of the time; ⑤ A little of the time; ⑥ Rarely; ⑦ Not at all

27. Over the past 2 weeks, how much time have you felt angry due to your bowel problems?

① All the time; ② Most of the time; ③ A lot of the time; ④ Some of the time; ⑤ A little of the time; ⑥ Rarely; ⑦ Not at all

28. Over the past 2 weeks, how much did bowel problems limit your sexual activity?

① No sexual activity due to bowel issues; ② Severely limited; ③ Moderately limited; ④ Slightly limited; ⑤ Minimally limited; ⑥ Rarely limited; ⑦ Not limited

29. Over the past 2 weeks, how much time have you been troubled by nausea or stomach discomfort?

① All the time; ② Most of the time; ③ A lot of the time; ④ Some of the time; ⑤ A little of the time; ⑥ Rarely; ⑦ Not at all

30. Over the past 2 weeks, how much time have you felt restless or easily annoyed?

① All the time; ② Most of the time; ③ A lot of the time; ④ Some of the time; ⑤ A little of the time; ⑥ Rarely; ⑦ Not at all

31. Over the past 2 weeks, how much time have you felt misunderstood by others?

① All the time; ② Most of the time; ③ A lot of the time; ④ Some of the time; ⑤ A little of the time; ⑥ Rarely; ⑦ Not at all

32. Over the past 2 weeks, how satisfied, happy, or content have you been with your personal life?

① Mostly very dissatisfied/unhappy; ② Generally dissatisfied/unhappy; ③ Somewhat dissatisfied/unhappy; ④ Generally satisfied/happy; ⑤Mostly satisfied/ happy; ⑥ Mostly very satisfied/happy; ⑦ Extremely satisfied/happy

**The Chinese version of the Symptom Checklist-90 (SCL-90).**

Below is a list of problems and complaints that people sometimes have. Please read each one carefully. After you have done so, select one of the numbered descriptors that best describes ‘how much that problem has bothered or distressed you during the past week, including today’. Circle the number in the space to the right of the problem and do not skip any items. Each item is scored on a scale from 0 to 4 based on how much an individual was bothered by each item in the last week: 0 = Not at all; 1 = A little bit; 2 = Moderately; 3 = Quite a bit; 4 = Extremely.

1. Headaches

2. Nervousness or shakiness inside

3. Unwanted thoughts, words, or ideas that won’t leave your mind

4. Faintness or dizziness

5. Loss of sexual interest or pleasure

6. Feeling critical of others

7. The idea that someone else can control your thoughts

8. Feeling others are to blame for most of your troubles

9. Trouble remembering things

10. Worried about sloppiness or carelessness

11. Feeling easily annoyed or irritated

12. Pains in heart or chest

13. Feeling afraid in open spaces or on the streets

14. Feeling low in energy or slowed down

15. Thoughts of ending your life

16. Hearing voices that other people do not hear

17. Trembling

18. Feeling that most people cannot be trusted

19. Poor appetite

20. Crying easily

21. Feeling shy or uneasy with the opposite sex

22. Feeling of being trapped or caught

23. Suddenly scared for no reason

24. Temper outbursts that you could not control

25. Feeling afraid to go out of your house alone

26. Blaming yourself for things

27. Pains in lower back

28. Feeling blocked in getting things done

29. Feeling lonely

30. Feeling blue

31. Worrying too much about things

32. Feeling no interest in things

33. Feeling fearful

34. Your feelings being easily hurt

35. Other people being aware of your private thoughts

36. Feeling others do not understand you or are unsympathetic

37. Feeling that people are unfriendly or dislike you

38. Having to do things very slowly to insure correctness

39. Heart pounding or racing

40. Nausea or upset stomach

41. Feeling inferior to others

42. Soreness of your muscles

43. Feeling that you are watched or talked about by others

44. Trouble falling asleep

45. Having to check and double-check what you do

46. Difficulty making decisions

47. Feeling afraid to travel on buses, subways, trains

48. Trouble getting your breath

49. Hot or cold spells

50. Having to avoid certain things, places, or activities because they frighten you

51. Your mind going blank

52. Numbness or tingling in parts of your body

53. A lump in your throat

54. Feeling hopeless about the future

55. Trouble concentrating

56. Feeling weak in parts of your body

57. Feeling tense or keyed up

58. Heavy feelings in your arms or legs

59. Thoughts of death or dying

60. Overeating

61. Feeling uneasy when people are watching or talking about you

62. Having thoughts that are not your own

63. Having urges to beat, injure, or harm someone

64. Awakening in the early morning

65. Having to repeat the same actions such as touching, counting, washing

66. Sleep that is restless or disturbed

67. Having urges to break or smash things

68. Having ideas or beliefs that others do not share

69. Feeling very self-conscious with others

70. Feeling uneasy in crowds, such as shopping or at a movie

71. Feeling everything is an effort

72. Spells of terror or panic

73. Feeling uncomfortable about eating or drinking in public

74. Getting into frequent arguments

75. Feeling nervous when you are left alone

76. Others not giving you proper credit for your achievements

77. Feeling lonely even when you are with people

78. Feeling so restless you couldn’t sit still

79. Feelings of worthlessness

80. Feeling that familiar things are strange or unreal

81. Shouting or throwing things

82. Feeling afraid you will faint in public

83. Feeling that people will take advantage of you if you let them

84. Having thoughts about sex that bother you a lot

85. The idea that you should be punished for your sins

86. Feeling pushed to get things done

87. The idea that something serious is wrong with your body

88. Never feeling close to another person

89. Feelings of guilt

90. The idea that something is wrong with your mind

**The Chinese version of the Social Support Rating Scale (SSRS).**

The following questions are used to reflect the support you receive in society. Please answer according to your actual situation as required for each question.

1. How many close friends do you have from whom you can get support or help (Choose only one option)?

A. None; B. 1 – 2; C. 3 – 5; D. 6 or more

2. In the past year, you ____ (Choose only one option)?

A. Have been away from your family; B. Have often changed your place of residence and mostly lived with strangers; C. Have lived with classmates, colleagues, or friends; D. Have lived with your family.

3. Regarding your relationship with your neighbors (Choose only one option)

A. You never care about each other and just nod in greeting; B. They may show a little concern if you encounter difficulties; C. Some neighbors care about you; D. Most neighbors care about you.

4. Regarding your relationship with your colleagues (Choose only one option)

A. You never care about each other and just nod in greeting; B. They may show a little concern if you encounter difficulties; C. Some colleagues care about you; D. Most colleagues care about you.

5. The support and care you receive from family members (Please fill in ①-④ in the brackets. ①-④ can be repeated)

① None ② Very little ③ Moderate ④ Full support

Spouse/Lover ( ); Parents ( ); Children ( ); Siblings ( ); Other members ( )

6. In the past, when you encountered urgent and difficult situations, the sources of financial or problem - solving help you received were (You can tick the options):

(1) No source at all;

(2) The following sources (Multiple options are available)

A. Spouse; B. Other family members; C. Friends; D. Relatives; E. Colleagues; F. Workplace

7. In the past, when you encountered urgent and difficult situations, the sources of comfort and concern you received were (You can tick the options):

(1) No source at all

(2) The following sources (Multiple options are available)

A. Spouse; B. Other family members; C. Friends; D. Relatives; E. Colleagues; F. Workplace; G. Official or semi-official organizations such as the Party, the League, and trade unions; H. Non-official organizations such as religious and social groups; I. Others (Please list).

8. Your way of confiding when you encounter troubles: (Choose only one option)

A. Don't confide in anyone; B. Only confide in 1 - 2 very close people; C. If a friend asks you proactively, you will talk about it; D. Proactively confide your troubles to get support and understanding.

9. Your way of seeking help when you encounter troubles (Choose only one option)

A. Rely only on yourself and don't accept help from others; B. Seldom ask for help from others; C. Sometimes ask for help from others; D. Often seek help from family, relatives, friends, and organizations when in trouble.

10. Regarding the activities organized by groups (such as Party organizations, religious organizations, trade unions, student unions, etc.), you (Choose only one option)

A. Never participate; B. Participate occasionally; C. Participate frequently; D. Actively participate and be active in the activities.
